# Supplementary material for: Mitochondrial Ca2+ Dynamics in MCU Knockout C. elegans Worms
Source: Int J Mol Sci. 2020 Nov 16;21(22):8622. doi: 10.3390/ijms21228622 (PMC7696937; doi:10.3390/ijms21228622)
Supplement: Supplementary file 1 [file ijms-21-08622-s001.pdf]

International Journal of
Molecular Sciences

Supplementary Materials

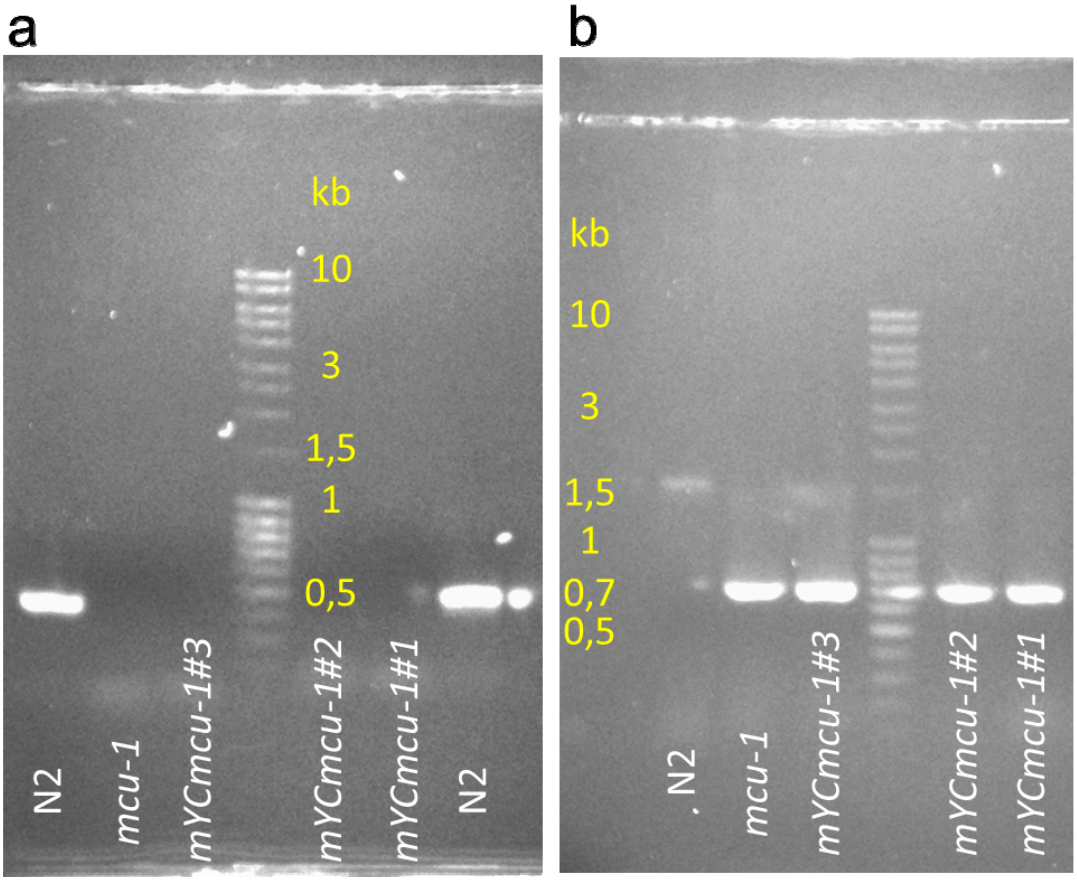

Figure S1. Genotyping of the *mcu-1* strain. Figure S1a shows the amplification of a PCR fragment placed inside the region of the *mcu-1* gene that has been deleted in the *mcu-1* strain. Only the N2 worms show the 454bp band, while the *mcu-1* strain and several *mYCmcu-1* strains (#1, #2 and #3) do not have it at all. Figure S1b shows the amplification of a PCR fragment that includes the region of the *mcu-1* gene that has been deleted in the *mcu-1* strain. The N2 worms show the full 1521 bp band, while *mcu-1* and *mYCmcu-1* strains show the truncated 700bp band.

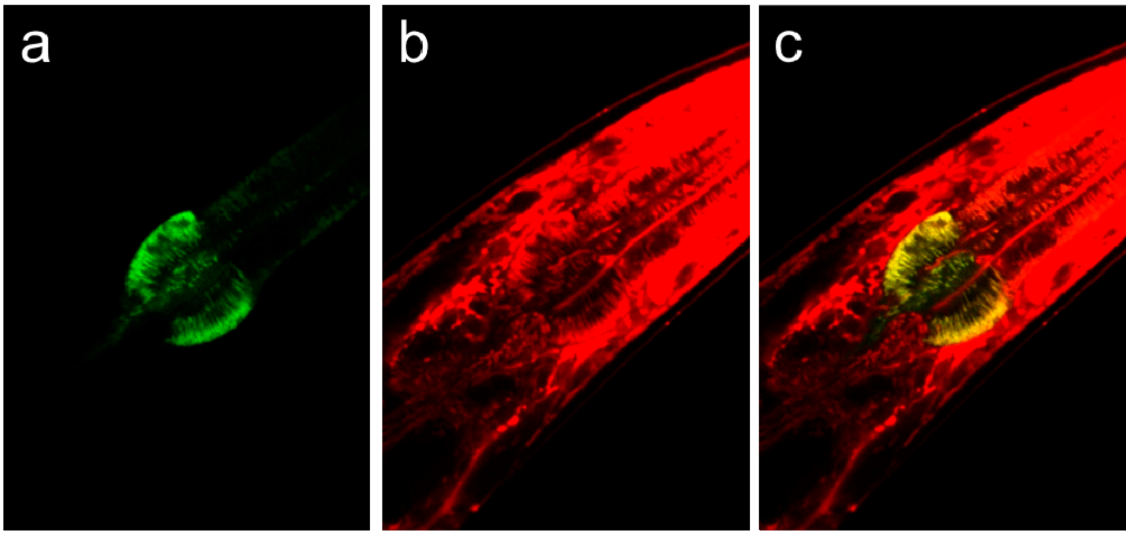

Figure S2. Confocal image showing the mitochondrial localization of the YC3.60 Ca^{2+} probe in the pharynx of the *mYCmcu-1* strain. The images show the YC3.60 fluorescence (left), the mitotracker deep red fluorescence (middle), and the merge of both (right).
